# Risk of T_2_ lesions when discontinuing fingolimod: a nationwide predictive and comparative study

**DOI:** 10.1093/braincomms/fcad358

**Published:** 2024-01-02

**Authors:** Malthe Faurschou Wandall-Holm, Rolf Pringler Holm, Alex Heick, Annika Reynberg Langkilde, Melinda Magyari

**Affiliations:** Department of Neurology, Danish Multiple Sclerosis Registry, Copenhagen University Hospital—Rigshospitalet, Glostrup DK-2600, Denmark; Department of Neurology, Danish Multiple Sclerosis Registry, Copenhagen University Hospital—Rigshospitalet, Glostrup DK-2600, Denmark; Department of Neurology, Danish Multiple Sclerosis Center, Copenhagen University Hospital—Rigshospitalet, Glostrup DK-2600, Denmark; Department of Radiology, Diagnostic Centre, Copenhagen University Hospital, Rigshospitalet, Copenhagen DK-2100, Denmark; Department of Neurology, Danish Multiple Sclerosis Registry, Copenhagen University Hospital—Rigshospitalet, Glostrup DK-2600, Denmark; Department of Neurology, Danish Multiple Sclerosis Center, Copenhagen University Hospital—Rigshospitalet, Glostrup DK-2600, Denmark

**Keywords:** multiple sclerosis, disease-modifying therapy, cohort study, treatment discontinuation, safety

## Abstract

Fingolimod is a frequently used disease-modifying therapy in relapsing–remitting multiple sclerosis. However, case reports and small observational studies indicate a highly increased risk of disease reactivation after discontinuation. We aimed to investigate the risk of radiological disease reactivation in patients discontinuing fingolimod. We performed a nationwide cohort study in Denmark, including patients who discontinued fingolimod between January 2014 and January 2023. Eligibility was a diagnosis with relapsing–remitting multiple sclerosis and two MRIs performed respectively within 1 year before and after discontinuing fingolimod. The included patients were compared with those discontinuing dimethyl fumarate with the same eligibility criteria in an unadjusted and matched propensity score analysis. Matching was done on age, sex, Expanded Disability Status Scale, MRI data, cause for treatment discontinuation, treatment duration and relapse rate. The main outcome was the presence of new T_2_ lesions on the first MRI after treatment discontinuation. To identify high-risk patients among those discontinuing fingolimod, we made a predictive model assessing risk factors for obtaining new T_2_ lesions. Of 1324 patients discontinuing fingolimod in the study period, 752 were eligible for inclusion [mean age (standard deviation), years, 41 (10); 552 females (73%); median Expanded Disability Status Scale (Q1–Q3), 2.5 (2.0–3.5); mean disease duration (standard deviation), years, 12 (8)]. Of 2044 patients discontinuing dimethyl fumarate in the study period, 957 were eligible for inclusion, presenting similar baseline characteristics. Among patients discontinuing fingolimod, 127 (17%) had 1–2 new T_2_ lesions, and 124 (17%) had ≥3 new T_2_ lesions compared with 114 (12%) and 45 (5%), respectively, for those discontinuing dimethyl fumarate, corresponding to odds ratios (95% confidence interval) of 1.8 (1.3–2.3) and 4.4 (3.1–6.3). The predictive model, including 509 of the 752 patients discontinuing fingolimod, showed a highly increased risk of new T_2_ lesions among those with disease activity during fingolimod treatment and among females under 40 years. This nationwide study suggests that discontinuing fingolimod in some cases carries a risk of developing new T_2_ lesions, emphasizing the importance of clinical awareness. If feasible, clinicians should prioritize the prompt initiation of new disease-modifying therapies, particularly among young females.

## Teaser

Wandall-Holm *et al*. demonstrated that discontinuing fingolimod, a frequently used treatment for multiple sclerosis, carries a significant risk of radiological disease reactivation. If feasible, clinicians should prioritize the prompt initiation of new disease-modifying therapies, particularly among young females.

## Graphical Abstract

**Graphical Abstract:**
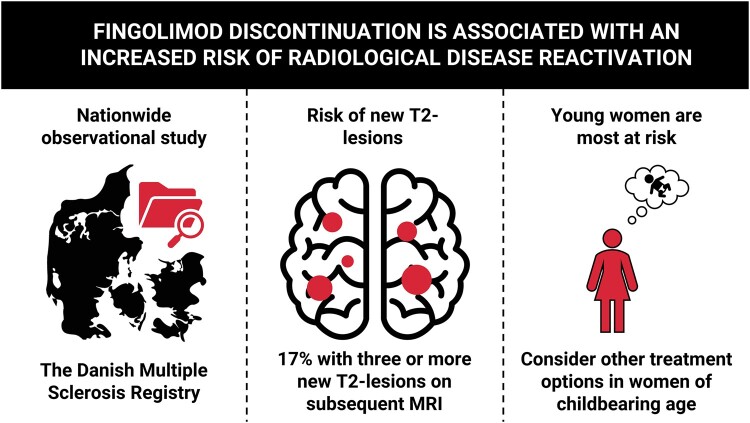

## Introduction

Fingolimod (FTY) was introduced in Europe in 2011 as the first oral disease-modifying therapy (DMT) for patients with relapsing–remitting multiple sclerosis (RRMS).^1^ It is a sphingosine-1-phosphate analogue interfering with a critical mechanism lymphocytes use to exit lymph nodes.^1,2^ This effect causes nodal trapping of lymphocytes and creates mild circulating lymphopenia preventing lymphocytes from entering the CNS to initiate multiple sclerosis (MS) lesions.^3^ When discontinuing FTY, thereby removing the trapping mechanism, patients could experience a rapid reentry of lymphocytes into the CNS, causing disease worsening.^3-5^ This theory corresponds with several case reports from the last decade showing patients experiencing dramatic increases in disease activity in the first few months after discontinuing FTY.^5-17^ Despite these case reports, studies have found conflicting evidence concerning the risk of a ‘rebound’ or reactivation phenomenon partly due to different definitions of the terminology.^3,18-23^

A recent study from the Danish Multiple Sclerosis Registry (DMSR) could not show clear evidence of clinical rebound based on relapse activity on a population level after FTY discontinuation among 992 patients with RRMS.^18^ However, case reports and clinical experience indicate that the sudden rise in disease reactivation is often seen on MRI measures.

We expect increased disease activity when discontinuing a DMT. To evaluate whether that seen for FTY differs from the typical pattern, we introduced a comparison. Dimethyl fumarate (DMF) is also an oral DMT showing a similar efficacy in phase III trials^24,25^ and head-to-head studies^26,27^ but without reported risk of rebound at discontinuation.

This study aimed to characterize the population discontinuing FTY and identify individuals at high risk of new T_2_ lesions (NT2L). Further, we wanted to investigate the risk of radiological disease reactivation when discontinuing FTY compared with DMF.

## Materials and methods

### Study design and population

We conducted a Danish nationwide study consisting of three parts: a descriptive cross-sectional study of patients discontinuing FTY treatment, an unmatched and propensity score–matched comparison between patients discontinuing FTY and DMF on the frequency of NT2L and a predictive model assessing the risk of NT2L. The cross-sectional study included all patients discontinuing FTY treatment between January 1, 2014, and January 1, 2023. Eligibility for study inclusion was a diagnosis with MS according to contemporary McDonald criteria (the 2010 revision from 2014 to 2017 and the 2017 revision from 2017 to the end of the study) and an RRMS phenotype at the date of treatment discontinuation, termed the baseline date. The patients had to have an MRI performed in the year leading up to the baseline date, termed ‘pre-MRI’, and an MRI completed within 1 year after the baseline date, termed ‘post-MRI’. The second part of the study contained a DMF comparison group with inclusion criteria identical to those in the cross-sectional study.

In the third, predictive part of the study, we applied additional restrictions to align the population with a typical clinical scenario and retain the ability to detect the formation of NT2L. Patients had to be between 18 and 55 years, have fewer than six NT2L on the pre-MRI, fewer than three relapses in the year before treatment discontinuation, a disease duration shorter than 30 years and a baseline Expanded Disability Status Scale score (EDSS) ≤ 4.

### Ethics, approvals and guidelines

The study was performed under the mandated rules by the Danish Data Protection Agency. Approval from an ethical committee and informed consent is not required in Denmark when performing anonymized observational register-based studies. Reporting of the study adhered to the Strengthening the Reporting of Observational Studies in Epidemiology^28^ (STROBE) recommendations.

### Data source, variables and outcomes

We retrieved data from the DMSR,^29^ a nationwide registry containing data on all patients with MS in Denmark. Clinicians enter data in 1 of the 13 MS clinics distributed around the country. The DMSR is a data provider to the Danish Clinical Registers^30^ in Denmark, which is mandated by law and regulated by the national government. The Danish Clinical Quality Program monitors the quality of MS treatments through data delivery via DMSR, ensuring high validity and completeness.

From the DMSR, we retrieved personal data on age, sex, diagnosis, phenotype, disease duration, EDSS scores, relapse activity, treatment and MRI. All baseline data were collected in the year leading up to the baseline date, retaining the latest value. We disregarded any EDSS occurring within 30 days of relapse to avoid relapse-related fluctuations. The post-MRI was the first MRI occurring after the baseline date within 1 year of treatment discontinuation.

Patients were categorized as having disease activity if the reason for treatment discontinuation was reported as disease activity or if the patient had either a relapse or NT2L reported in the year leading up to the baseline date.

For the predictive model, the outcome was having ≥1 NT2L on the post-MRI. For the comparative model, the outcome was NT2L on post-MRI grouped into 0, 1–2 and ≥3.

### Magnetic resonance imaging

In Denmark, all patients with MS are scanned at radiological departments servicing MS clinics by specialized neuroradiologist. Data from the radiological reports are subsequently entered into the DMSR. Information on total number of T_2_ lesions and number of new or enlarging lesions and number of enhancing lesions are registered. The NT2L outcome used in this study is a composite outcome containing both new and enlarging lesions. The Danish Society of Neuroradiology (DSNR) has recommended an MRI protocol in accordance with the one proposed by the MAGNIMS group.^31^ The required sequences include fluid-attenuated inversion recovery (FLAIR), T_1_ and T_2_, ideally as a 3D acquisition if possible. Gadolinium enhancement should be considered as well. In Denmark, the use of artificial intelligence image recognition software is not part of daily clinical practice. The intensity of the magnetic field is either 1.5 or 3 T depending on availability at the radiological site. Ideally, the same MRI machine is used for follow-ups. However, the data originate from clinical practice, and scanner malfunctions or patient relocations can necessitate the use of different scanners in this study. According to guidelines by the DSNR, new lesions are reported with a presence of new T_2_ hyperintense areas more than 3 mm with no better explanation (e.g. infarction), and an increase in an existing lesion of more than 50% is classified as enlarging. In the presence of confluencing lesions, a further confluencing lesion compared with the previous MRI will count as an enlarging lesion.

### Statistical analysis

Population characteristics were reported as means with standard deviations, medians with first and third quartile or number with percentages as appropriate.

We performed a multivariable logistic regression for the predictive model, including sex, EDSS, NT2L on pre-MRI as categorical covariates and age as a continuous covariate. We included an interaction term between sex and age due to the different disease courses between sexes. Age did not display a linear relationship with the log odds, and we introduced a linear spline effect at the age of 38. The model was done on a complete case basis.

In the comparative study, we calculated predicted probabilities with 95% confidence intervals and odds ratios (OR) using a multinomial logistic regression model with a generalized logit function. To mitigate indication bias, we also did propensity score–based matching with fixed nearest neighbor-matching, a ratio of 1:1 and a caliper of 0.1 standard deviations of the logit-transformed propensity score. Matching variables were age, sex, EDSS, number of NT2L on pre-MRI, cause for treatment discontinuation, treatment duration, number of relapses the year before discontinuation and an interaction between age and sex. Balance was assessed using standardized difference, and a difference below 0.1 was considered acceptable.

T_2_ lesion count has demonstrated prognostic value in predicting the future course of MS.^32,33^ Not accounting for T_2_ lesion count status in the matching process could introduce potential bias. To mitigate this, we performed a sensitivity analysis in which we also matched subjects based on a categorization of T_2_ lesion count: 0, 1–19 or more than 20 lesions. Another potential determinant of disease activity and formation of NT2L following treatment discontinuation is the initiation of a new DMT before the post-MRI. We, therefore, performed another sensitivity analysis in which we additionally matched subjects on two categorical covariates, new DMT—none, moderate or high efficacy—and time to new DMT from original treatment discontinuation (none, ≤30 days or >30 days).

Data management and statistical analyses were performed using SAS software, version 9.4 (SAS Institute Inc., Cary, NC, USA).

## Results

### Cross-sectional study

**Figure 1:**
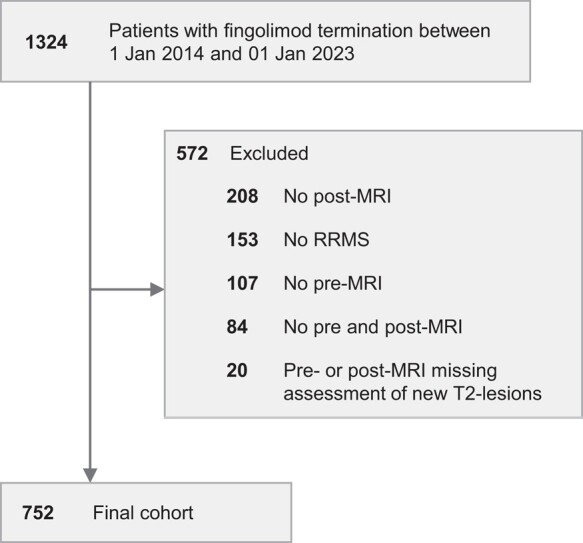
**Flow diagram—fingolimod**. RRMS, relapsing–remitting multiple sclerosis.

We identified 1324 patients who discontinued FTY in the study period, of which 752 were eligible for inclusion in the cross-sectional study (Fig. 1).

The baseline characteristics are illustrated in Table 1. In total, 524 (69.7%) discontinued FTY with disease activity and 228 (30.3%) without disease activity the previous year. The groups had roughly similar sex distribution, disease duration, EDSS at baseline and treatment duration. However, they differed in age, with the no-disease activity group being older (44.2 versus 40.2 years) and having a longer time since the pre-MRI (145 versus 85 days). In the group with no disease activity, the majority stopped due to adverse events related to the treatment, 154 (67.5%).

**Table 1:** Clinical and demographic characteristics

	Disease activity^a^	No disease activity^a^
Patients, no.	524	228
Age at baseline, years, mean (SD)	40.2 (10.3)	44.2 (10.5)
Sex, no. (%)		
Female	384 (73.3)	168 (73.7)
Male	140 (26.7)	60 (26.3)
Disease duration, years, mean (SD)	11.2 (7.3)	13.4 (7.9)
EDSS, median (Q1–Q3)	2.5 (2.0–3.5), *n*_miss_ = 28	2.5 (1.5–3.5), *n*_miss_ = 10
Number of relapses in the past year, mean (SD)	1.0 (0.8)	—
Fingolimod treatment duration, years, median (Q1–Q3)	2.3 (0.8–4.4)	2.4 (1.1–4.7)
Days since pre-MRI, days, median (Q1–Q3)	85 (43–168)	145 (68–255)
Recorded cause for fingolimod discontinuation, no. (%)		
Disease activity	374 (71.4)	—
Adverse events	110 (21.0)	154 (67.5)
Other^b^	27 (5.2)	43 (18.9)
Pregnancy wish	13 (2.5)	31 (13.6)

^a^Status was defined as ‘disease activity’ if the patient met one of the following criteria: fingolimod discontinuation due to disease activity, one or more relapses in the last year before fingolimod discontinuation or the presence of new T_2_ lesion(s) on the last MRI in the last year before fingolimod discontinuation.

^b^Listed as other: lack of patient compliance, practical issues, stable condition, contra-indication or patient decision.

EDSS, Expanded Disability Status Scale.

Of the 752 patients, 127 (16.9%) had 1–2 NT2L, and 124 (16.5%) had ≥3 NT2L on the post-MRI. We divided the population into three groups according to the time from FTY discontinuation to the post-MRI (Table 2). For patients with disease activity, we see a uniform distribution of NT2L in all three groups, with ∼60% showing zero lesions, 20% 1–2 lesions and 20% having ≥3 on the post-MRI. The no-disease activity group varied more, having a post-MRI in the first 3 months displaying a lower NT2L count with 80% having zero lesions, compared with the two latter groups, where ∼70% had zero lesions.

**Table 2:** Post-MRI characteristics

Time since fingolimod discontinuation	1–3 months		4–6 months		7–12 months		1–12 months	
Disease activity^a^	−	+	−	+	−	+	−	+
Patients, no.	97	224	52	123	79	177	228	524
New T_2_ lesions on post-MRI, grouped, no. (%)								
0	78 (80.4)	140 (62.5)	36 (69.2)	74 (60.2)	58 (73.4)	115 (65.0)	172 (75.4)	329 (62.8)
1–2	15 (15.5)	43 (19.2)	5 (9.6)	23 (18.7)	11 (13.9)	30 (16.9)	31 (13.6)	96 (18.3)
≥3	4 (4.1)	41 (18.3)	11 (21.2)	26 (21.1)	10 (12.7)	32 (18.1)	25 (11.0)	99 (18.9)
Days since pre-MRI, median (Q1–Q3)	226 (160–323)	158 (98–247)	269 (176–368)	207 (172–277)	355 (269–431)	307 (243–376)	292 (204–365)	223 (155–315)
New T_2_ lesions on pre-MRI, median (Q1–Q3)	0 (0–0)	1 (0–2)	0 (0–0)	1 (0–2)	0 (0–0)	1 (0–2)	0 (0–0)	1 (0–2)

^a^Status was defined as ‘disease activity’ if the patient met one of the following criteria: fingolimod discontinuation due to disease activity, one or more relapses in the last year before fingolimod discontinuation or the presence of new T_2_ lesion(s) on the last MRI in the last year before fingolimod discontinuation.

We subdivided patients into those who received a new DMT before the post-MRI and those who remained untreated at the post-MRI. Unsurprisingly, patients with the longest treatment duration with a new DMT had a longer delay between FTY discontinuation and post-MRI. The 7–12 month group, with the most treatment coverage, showed a decrease in the occurrence of ≥1 NT2L from 46.5% in the 1–3 month group compared with 30.1% in the 7–12 month group. Conversely, the untreated displayed a rise from 29.9% in the 1–3 month group to 42.6% in the 7–12 month group having ≥1 NT2L (Supplementary Table 1).

Of the entire population, 687 (91.4%) started a new treatment within 1 year. The median time from FTY discontinuation to start of a new treatment was 50 days (Q1–Q3: 34–83) and remained at approximately the same level throughout the study period (Supplementary Fig. 1). The median time from FTY discontinuation to the post-MRI was 111 days (Q1–Q3: 40.5–195) with considerable variation between individuals. There was some fluctuation between calendar years, but the duration remained unchanged overall (Supplementary Fig. 2).

### Comparison

We identified 2044 patients who discontinued DMF in the study period, of which 957 were eligible for inclusion in the comparison study (Supplementary Fig. 3). The unmatched populations had similar baseline characteristics but showed differences in disease duration (FTY: 11.9 years versus DMF: 8.4 years) and treatment duration (FTY: 2.3 years versus DMF: 1.0 years). Additionally, the FTY population had 49.7% discontinuing treatment due to disease activity versus 40.2% among the DMF population. In the propensity score–matched population, the differences were decreased markedly. For all baseline characteristics, see Supplementary Table 2.

Table 3 displays the prevalence of NT2L on the post-MRI divided into three categories: 0, 1–2 and ≥3. The unmatched and the matched population showed almost identical NT2L distributions within drug groups. The FTY population had significantly higher odds of NT2L on the post-MRI than the DMF population, being approximately twice as high for 1–2 NT2L and four to five times as high for ≥3 NT2L. In both the matched and unmatched FTY population, ∼17% experienced 1–2 NT2L and 17% ≥3 NT2L.

**Table 3:** Frequency of new T_2_ lesions on post-MRI

	Fingolimod	Probability (95% CI)^a^	Dimethyl fumarate	Probability (95% CI)^a^	Odds ratio^b^
Unmatched					
Patients, no.	752		957		
New T_2_ lesions, no.					
0	501		798		
1–2	127	16.9% (14.2–19.6)	114	11.9% (9.9–14.0)	1.8 (1.3–2.3)^c^
≥3	124	16.5% (13.8–19.1)	45	4.7% (3.4–6.0)	4.4 (3.1–6.3)^c^
Matched^d^					
Patients, no.	527		527		
New T_2_ lesions, no.					
0	343		444		
1–2	96	18.2% (14.9–21.5)	60	11.4% (8.7–14.1)	2.1 (1.5–2.9)^c^
≥3	88	16.7% (13.5–19.9)	23	4.4% (2.6–6.1)	5.0 (3.1–8.0)^c^

^a^Predictions from the multinominal logistic regression, see Materials and methods section.

^b^Fingolimod versus dimethyl fumarate.

^c^ *P* < 0.001.

^d^Propensity score matched based on age, sex, Expanded Disability Status Scale, number of new T_2_ lesions on pre-MRI, cause for termination of treatment, treatment duration, number of relapses the past year and an interaction between age and sex.

The sensitivity analyses including lesion count and information on new DMT initiation confirmed findings in the main analysis (for baseline characteristics, see Supplementary Table 2, and for prevalence of NT2L, see Supplementary Table 4).

### Prediction

**Figure 2:**
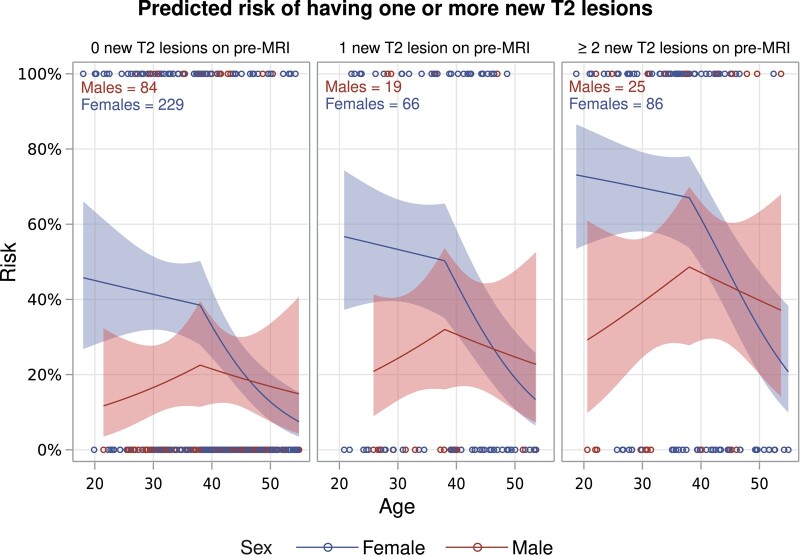
**Predicted risk of one or more new T_2_ lesions on post-MRI.** The plots are divided by the number of new T_2_ lesions on pre-MRI and display the absolute predicted risk of having one or more new T_2_ lesions on the post-MRI according to sex and age. Transparent areas represent 95% confidence intervals. The graphs shown are for an EDSS score of 2–3.5 (48.8% of the population). Hollow circles represent individuals. The statistical analysis performed to produce the plots was a logistic regression with a spline effect on age and an interaction term between the age spline and sex. The experimental unit was participants in the study. Pre-MRI: the last MRI prior to fingolimod discontinuation. Post-MRI: the first MRI post fingolimod discontinuation. EDSS, Expanded Disability Status Scale. Wald Chi-square (degrees of freedom, *P*-value): sex, 3.28 (1, 0.07); age spline, 6.55 (2, 0.04); ages spline * sex interaction, 7.08 (2, 0.03); Expanded Disability Status Scale, 3.71 (2, 0.16); new T_2_ lesions on pre-MRI, 23.3 (2, <0.01); area under the curve (AUC), 0.73.

Of the original 752 patients discontinuing FTY from the cross-sectional study, 509 were included in the final model (excluded: 211 not meeting the additional inclusion criteria and 32 missing a baseline EDSS). The odds of having ≥1 NT2L on post-MRI increased by 225% [OR: 3.25, 95% confidence interval (CI): 2.01–5.27, *P* < 0.001] if the patient had ≥2 NT2L on the pre-MRI and 62% (OR: 1.62, 95% CI: 0.95–2.76, *P* = 0.08) if the patient had one NT2L on the pre-MRI compared with zero lesions. The description of the risk profile with regard to sex and age with an interaction term and spline effect is best depicted in Fig. 2. The figures display different risk profiles for males and females. Females were at the highest risk below 40 years, decreasing hereafter, whereas the effect of age on males was much less pronounced and displayed a slight risk increase with increasing age. For both sexes, there is a marked increase in risk with an increasing number of NT2L on the pre-MRI.

## Discussion

In this Danish nationwide study, we investigated radiological disease reactivation among patients discontinuing FTY treatment between 2014 and 2023. Patients were on average 40 years with a female-to-male ratio of 3:1 and a median EDSS at discontinuation of 2.5. In the year leading up to treatment discontinuation, 70% displayed disease activity. On the first post-MRI, 37% of those with disease activity and 25% of those without disease activity experienced ≥1 NT2L. The study design did not allow for a direct causal interpretation of the protective effect of starting a new DMT. However, we did display an inverse relationship between increasing duration of treatment and fewer NT2L on the post-MRI. Patients discontinuing FTY had a much higher absolute risk of NT2L on the post-MRI compared with DMF, both in the category of 1–2 NT2L (17% versus 12%) and highly pronounced in the category ≥3 (17% versus 5%). Our predictive model for those discontinuing FTY showed that having NT2L on the pre-MRI and being female significantly increased the risk of NT2L on the post-MRI. Age did not greatly affect the risk among males, whereas age had a sizeable two-part effect on females, being very high below 40 years (40–70%) and declining sharply hereafter.

Comparisons with other studies are challenging due to various study designs, outcome measures and clinical reactivation definitions. We found MRI activity among a third of the patients after FTY discontinuation, higher than seen in studies looking into clinical rebound^18,20^ and a *post hoc* analysis made on the available MRI data from the initial phase III studies looking at MRI outliers, 17/300 (5.7%).^23^ On the other hand, our findings align with secondary outcomes from a study finding 62/116 (53.4%) patients experiencing new MRI activity after FTY discontinuation^34^ and a small study finding MRI activity among 10/19 (52.6%) patients.^21^ The discrepancy between studies evaluating clinical relapses and MRI findings could be due to the increased sensitivity of MRI and low relapse detection rates.

Compared with DMF discontinuation, we found a statistically significant increased risk of NT2L, which could raise concerns about initiating patients on FTY. However, another study did not find a statistically significant difference in new gadolinium-enhancing lesions when comparing FTY and DMF 6–12 months after discontinuation.^35^ According to current evidence, disease reactivation typically occurs 4–16 weeks after FTY discontinuation,^12-14,16,17,20,36^ and the study only had MRI data available on 24% of the included population. As such, their study might have missed a critical window when evaluating discontinuation-related MRI activity and introduced a potential selection bias. In comparison, in our study, 75% of patients had a follow-up MRI during the first 6 months.

Our results showed a higher risk of NT2L when discontinuing FTY due to disease activity, underlining the importance of quick initiation of a new DMT in this subgroup of patients. Furthermore, the predictive model showed a significantly higher risk of NT2L among young females. Our findings support our research group’s previous study on a clinical rebound, which indicated a greater risk of disease reactivation among younger females and those discontinuing due to disease activity. However, our study showed an even higher risk when using radiological activity as an outcome measure. To our knowledge, we are the first study investigating the age–sex interaction, but other studies have found younger age to be a risk factor as well.^20,22,34,37^ In females with a pregnancy wish, exploring other treatment avenues to avoid potential cases with discontinuation followed by severe disease activation in the vulnerable period during or after pregnancy should be considered.

Due to clinical experiences and case reports of disabling disease activity after FTY discontinuation, we expected to see a change in practice to expedite the initiation of a new DMT in this patient group. Surprisingly, the delay from FTY to the initiation of a new DMT did not change during the study period.

The study had several limitations. Firstly, the study was subject to selection bias due to the necessity of two MRIs within a short period of time. This requirement resulted in the exclusion of 419 (32%) patients in the FTY population and 963 (50%) in the DMF population; baseline characteristics can be found in Supplementary Table 3. Patients with less disease activity, who are less likely to undergo frequent MRIs due to their stable conditions, may be disproportionately excluded. Consequently, our study results may overestimate the disease activity in patients discontinuing FTY or DMF, as the included patients might represent a subset with higher disease activity than typically observed in the whole population discontinuing these treatments. Secondly, there is a temporal bias. We expect clinicians to expedite MRI scans and initiation of new DMTs in those identified as high-risk patients. As such, comparison of a patient with an MRI shortly after FTY discontinuation and one with an MRI after a year could be biased. To reduce this bias, we divided the year into three categories, allowing for comparison of more temporally similar patients. Thirdly, in our predictive model, we did introduce a spline due to the non-linear effect of age. We did not expect this relationship *a priori*. Adjusting the model after seeing the data could lead to overfitting, reducing generalizability. Fourthly, we chose DMF as the comparator. However, the choice of DMF could introduce an indication bias since DMF is considered a first-line therapy and FTY a second-line therapy in Denmark. This bias seemed negligible; when comparing baseline characteristics, both groups appeared quite similar, and applying propensity score matching did not change results. Fifth, awareness of the reactivation phenomenon could guide clinicians to change clinical practice over time, introducing a calendar effect not accounted for in the study. However, looking at the time from FTY discontinuation to the initiation of a new DMT or a new MRI scan, the duration remained stationary during the study period. Sixth, lesions on the post-MRI might have appeared before FTY discontinuation since the median period from pre-MRI until treatment discontinuation was 98 days (Q1–Q3: 45–193). Hence, new lesions might occur during treatment due to treatment failure and not because of a reactivation phenomenon. Seventh, confluencing lesions can present a challenge when attempting to count the exact number of T_2_ lesions. This study only applies lesion count in a sensitivity analysis, categorizing lesions into 0, 1–19 and more than 20. As the issue of counting exactly primarily affects patients with over 20 lesions, it’s of minimal concern in this context.

Our study’s strengths were access to nationwide coverage of the MS population, free health care ensuring equal access and high data completeness enabling the application of strict MRI inclusion criteria of two MRI scans within 2 years.

In conclusion, our nationwide study suggests that discontinuing fingolimod in some cases carries a risk of developing NT2L, emphasizing the importance of clinical awareness. If feasible, clinicians should prioritize the prompt initiation of new DMTs, particularly among young females.

## Supplementary material

Supplementary material is available at *Brain Communications* online.

## Supplementary Material

fcad358_Supplementary_Data

## Data Availability

The study was approved by the Danish Data Protection Agency. Danish data regulations dictate that access to data can only be obtained upon qualified request and approval by the Danish Data Protection Agency and the Danish Multiple Sclerosis Group.

## References

[1] European Medicines Agency . Gilenya—EMEA/H/C/002202—IA/0080. Published online 2022:28. https://www.ema.europa.eu/en/documents/product-information/gilenya-epar-product-information_en.pdf

[2] Brinkmann V . FTY720 (fingolimod) in multiple sclerosis: Therapeutic effects in the immune and the central nervous system. Br J Pharmacol. 2009;158(5):1173–1182. 19814729 10.1111/j.1476-5381.2009.00451.x PMC2782328

[3] Barry B, Erwin AA, Stevens J, Tornatore C. Fingolimod rebound: A review of the clinical experience and management considerations. Neurol Ther. 2019;8(2):241–250. 31677060 10.1007/s40120-019-00160-9 PMC6858914

[4] Cavone L, Felici R, Lapucci A, et al Dysregulation of sphingosine 1 phosphate receptor-1 (S1P1) signaling and regulatory lymphocyte-dependent immunosuppression in a model of post-fingolimod MS rebound. Brain Behav Immun. 2015;50:78–86. 26130058 10.1016/j.bbi.2015.06.019

[5] Berger B, Baumgartner A, Rauer S, et al Severe disease reactivation in four patients with relapsing–remitting multiple sclerosis after fingolimod cessation. J Neuroimmunol. 2015;282:118–122. 25903738 10.1016/j.jneuroim.2015.03.022

[6] Ghezzi A, Rocca MA, Baroncini D, et al Disease reactivation after fingolimod discontinuation in two multiple sclerosis patients. J Neurol. 2013;260(1):327–329. 23161460 10.1007/s00415-012-6744-7

[7] Sánchez P, Meca-Lallana V, Vivancos J. Tumefactive multiple sclerosis lesions associated with fingolimod treatment: Report of 5 cases. Mult Scler Relat Disord. 2018;25(July):95–98. 30056362 10.1016/j.msard.2018.07.001

[8] Hakiki B, Portaccio E, Giannini M, Razzolini L, Pastò L, Amato MP. Withdrawal of fingolimod treatment for relapsing–remitting multiple sclerosis: Report of six cases. Mult Scler J. 2012;18(11):1636–1639. 10.1177/1352458512454773 22829326

[9] Lapucci C, Baroncini D, Cellerino M, et al Different MRI patterns in MS worsening after stopping fingolimod. Neurol Neuroimmunol Neuroinflamm. 2019;6(4):e566. 31086807 10.1212/NXI.0000000000000566 PMC6481223

[10] Sempere AP, Berenguer-Ruiz L, Feliu-Rey E. Rebound of disease activity during pregnancy after withdrawal of fingolimod. Eur J Neurol. 2013;20(8):e109–e110. 23829238 10.1111/ene.12195

[11] La Mantia L, Prone V, Marazzi MR, Erminio C, Protti A. Multiple sclerosis rebound after fingolimod discontinuation for lymphopenia. Neurol Sci. 2014;35(9):1485–1486. 24756193 10.1007/s10072-014-1800-y

[12] Beran RG, Hegazi Y, Schwartz RS, Cordato DJ. Rebound exacerbation multiple sclerosis following cessation of oral treatment. Mult Scler Relat Disord. 2013;2(3):252–255. 25877732 10.1016/j.msard.2012.11.001

[13] Havla JB . Rebound of disease activity after withdrawal of fingolimod (FTY720) treatment. Arch Neurol. 2012;69(2):262. 22332194 10.1001/archneurol.2011.1057

[14] Hatcher SE, Waubant E, Nourbakhsh B, Crabtree-Hartman E, Graves JS. Rebound syndrome in patients with multiple sclerosis after cessation of fingolimod treatment. JAMA Neurol. 2016;73(7):790. 27135594 10.1001/jamaneurol.2016.0826

[15] Członkowska A, Smoliński Ł, Litwin T. Severe disease exacerbations in patients with multiple sclerosis after discontinuing fingolimod. Neurol Neurochir Pol. 2017;51(2):156–162. 28209440 10.1016/j.pjnns.2017.01.006

[16] Fragoso YD, Adoni T, Gomes S, et al Severe exacerbation of multiple sclerosis following withdrawal of fingolimod. Clin Drug Investig. 2019;39(9):909–913. 10.1007/s40261-019-00804-6 31152369

[17] Gündüz T, Kürtüncü M, Eraksoy M. Severe rebound after withdrawal of fingolimod treatment in patients with multiple sclerosis. Mult Scler Relat Disord. 2017;11:1–3. 28104247 10.1016/j.msard.2016.11.003

[18] Framke E, Pontieri L, Bramow S, Sellebjerg F, Magyari M. Rebound of clinical disease activity after fingolimod discontinuation? A nationwide cohort study of patients in Denmark. J Neurol Neurosurg Psychiatry. 2022;93:1317–1321. 10.1136/jnnp-2022-329607 36171103

[19] Barboza A, Gaitán MI, Alonso R, et al Rebound activity after fingolimod cessation: A case-control study. Mult Scler Relat Disord. 2022;57:103329. 35158443 10.1016/j.msard.2021.103329

[20] Goncuoglu C, Tuncer A, Bayraktar-Ekincioglu A, et al Factors associated with fingolimod rebound: A single center real-life experience. Mult Scler Relat Disord. 2021;56:103278. 34655957 10.1016/j.msard.2021.103278

[21] Sato K, Niino M, Kawashima A, Yamada M, Miyazaki Y, Fukazawa T. Disease exacerbation after the cessation of fingolimod treatment in Japanese patients with multiple sclerosis. Intern Med. 2018;57(18):2647–2655. 29709955 10.2169/internalmedicine.0793-18 PMC6191590

[22] Cerdá-Fuertes N, Nagy S, Schaedelin S, et al Evaluation of frequency, severity, and independent risk factors for recurrence of disease activity after fingolimod discontinuation in a large real-world cohort of patients with multiple sclerosis. Ther Adv Neurol Disord. 2023;16(6):175628642211503. 10.1177/17562864221150312 PMC9905031 36762317

[23] Vermersch P, Radue EW, Putzki N, Ritter S, Merschhemke M, Freedman MS. A comparison of multiple sclerosis disease activity after discontinuation of fingolimod and placebo. Mult Scler J Exp Transl Clin. 2017;3(3):2055217317730096. 28989795 10.1177/2055217317730096 PMC5624444

[24] Fox RJ, Miller DH, Phillips JT, et al Placebo-controlled phase 3 study of oral BG-12 or glatiramer in multiple sclerosis. N Engl J Med. 2012;367(12):1087–1097. 22992072 10.1056/NEJMoa1206328

[25] Gold R, Kappos L, Arnold DL, et al Placebo-controlled phase 3 study of oral BG-12 for relapsing multiple sclerosis. N Engl J Med. 2012;367(12):1098–1107. 22992073 10.1056/NEJMoa1114287

[26] Hersh CM, Love TE, Cohn S, et al Comparative efficacy and discontinuation of dimethyl fumarate and fingolimod in clinical practice at 12-month follow-up. Mult Scler Relat Disord. 2016;10:44–52. 27919497 10.1016/j.msard.2016.08.002

[27] Vollmer BL, Nair K V, Sillau S, Corboy JR, Vollmer T, Alvarez E. Natalizumab versus fingolimod and dimethyl fumarate in multiple sclerosis treatment. Ann Clin Transl Neurol. 2019;6(2):252–262. 30847358 10.1002/acn3.700 PMC6389745

[28] von Elm E, Altman DG, Egger M, Pocock SJ, Gøtzsche PC, Vandenbroucke JP. The Strengthening the Reporting of Observational Studies in Epidemiology (STROBE) statement: Guidelines for reporting observational studies*. Bull World Health Organ. 2007;85(11):867–872. 18038077 10.2471/BLT.07.045120 PMC2636253

[29] Magyari M, Joensen H, Laursen B, Koch-Henriksen N. The Danish Multiple Sclerosis Registry. Brain Behav. 2021;11(1):1–10. 10.1002/brb3.1921 PMC7821574 33128351

[30] Sørensen HT, Pedersen L, Jorgensen J, Ehrenstein V. Danish clinical quality databases—An important and untapped resource for clinical research. Clin Epidemiol. 2016;8:425–427. 27843338 10.2147/CLEP.S113265 PMC5098512

[31] Wattjes MP, Ciccarelli O, Reich DS, et al 2021 MAGNIMS–CMSC–NAIMS consensus recommendations on the use of MRI in patients with multiple sclerosis. Lancet Neurol. 2021;20(8):653–670. 34139157 10.1016/S1474-4422(21)00095-8

[32] Louapre C, Bodini B, Lubetzki C, Freeman L, Stankoff B. Imaging markers of multiple sclerosis prognosis. Curr Opin Neurol. 2017;30(3):231–236. 28362719 10.1097/WCO.0000000000000456

[33] Andravizou A, Dardiotis E, Artemiadis A, et al Brain atrophy in multiple sclerosis: Mechanisms, clinical relevance and treatment options. Autoimmun Highlights. 2019;10(1):7. 10.1186/s13317-019-0117-5 PMC7065319 32257063

[34] Landi D, Signori A, Cellerino M, et al What happens after fingolimod discontinuation? A multicentre real-life experience. J Neurol. 2022;269(2):796–804. 34136943 10.1007/s00415-021-10658-8

[35] Vollmer B, Ontaneda D, Harris H, et al Comparative discontinuation, effectiveness, and switching practices of dimethyl fumarate and fingolimod at 36-month follow-up. J Neurol Sci. 2019;407:116498. 31644992 10.1016/j.jns.2019.116498

[36] Francis G, Kappos L, O’Connor P, et al Temporal profile of lymphocyte counts and relationship with infections with fingolimod therapy. Mult Scler J. 2014;20(4):471–480. 10.1177/1352458513500551 23950550

[37] Pantazou V, Pot C, Du Pasquier R, Le Goff G, Théaudin M. Recurrence of disease activity after fingolimod discontinuation in older patients previously stable on treatment. Mult Scler Relat Disord. 2021;51:102918. 33838521 10.1016/j.msard.2021.102918

