# Supplementary material for: Risk of T2 lesions when discontinuing fingolimod: a nationwide predictive and comparative study
Source: Brain Commun. 2024 Jan 2;6(1):fcad358. doi: 10.1093/braincomms/fcad358 (PMC10783644; doi:10.1093/braincomms/fcad358)
Supplement: fcad358_Supplementary_Data [file fcad358_supplementary_data.docx]

**Supplementary Materials**

Malthe Faurschou Wandall-Holm^†^, Rolf Pringler Holm^†^, Alex Heick, Annika Reynberg Langkilde, Melinda Magyari.

^†^These authors contributed equally to this work.

**Supplementary Table 1.** Status at post-MRI, stratified by time from fingolimod discontinuation to post-MRI, subdivided into those receiving a new disease-modifying therapy at post-MRI and those remaining untreated.

**Supplementary Figure 1.** Days from fingolimod discontinuation to initiation of a new disease-modifying therapy.

**Supplementary Figure 2.** Days from fingolimod discontinuation to performance of the post-MRI.

**Supplementary Figure 3.** Flow Diagram – dimethyl fumarate.

**Supplementary Table 2.** Baseline characteristics, unmatched, propensity score-matched and sensitivity analysis, fingolimod vs. dimethyl fumarate.

**Supplementary Table 3.** Baseline characteristics of patients excluded due to missing pre-MRI, post-MRI or both pre- and post-MRI.

**Supplementary Table 4.** Frequency of new T2-lesions on post-MRI, sensitivity analysis.

**Supplementary Table 1.** Status at post-MRI, stratified by time from fingolimod discontinuation to post-MRI, subdivided into those receiving a new disease-modifying therapy at post-MRI and those remaining untreated.

|  | | **1-3 months** | | **4-6 months** | | **7-12 months** | | **1-12 months** | |
| --- | --- | --- | --- | --- | --- | --- | --- | --- | --- |
| **Initiation of new DMT** | | **-** | **+** | **-** | **+** | **-** | **+** | **-** | **+** |
| Patients, No. | | 278 | 43 | 48 | 127 | 47 | 209 | 373 | 379 |
| New T2-lesions on post-MRI, grouped, No. (%) | |  |  |  |  |  |  |  |  |
|  | 0 | 195 (70.1) | 23 (53.5) | 28 (58.3) | 82 (64.6) | 27 (57.4) | 146 (69.9) | 250 (67.0) | 251 (66.2) |
|  | ≥1 | 83 (29.9) | 20 (46.5) | 20 (41.7) | 45 (35.4) | 20 (42.6) | 63 (30.1) | 123 (33.0) | 128 (33.8) |
| Days on DMT at post-MRI, median (Q1-Q3) | | - | 14 (7-29) | - | 85 (63-108) | - | 175 (147-216) | - | 128 (77-178) |
| New T2-lesions on pre-MRI, median (Q1-Q3) | | 0 (0-1) | 0 (0-2) | 0 (0-0) | 0 (0-2) | 0 (0-0) | 0 (0-1) | 0 (0-1) | 0 (0-1) |
| Number of relapses in the last year before fingolimod discontinuation, mean (SD) | | 0.7 (0.8) | 0.7 (0.9) | 0.5 (0.7) | 0.6 (0.7) | 0.4 (0.7) | 0.7 (0.9) | 0.7 (0.8) | 0.7 (0.8) |

“-“ is no initiation of a new DMT at the post-MRI, “+” is initiation of a new DMT at the post-MRI.
DMT: disease-modifying therapy.

**Supplementary Figure 1.** Days from fingolimod discontinuation to initiation of a new disease-modifying therapy.


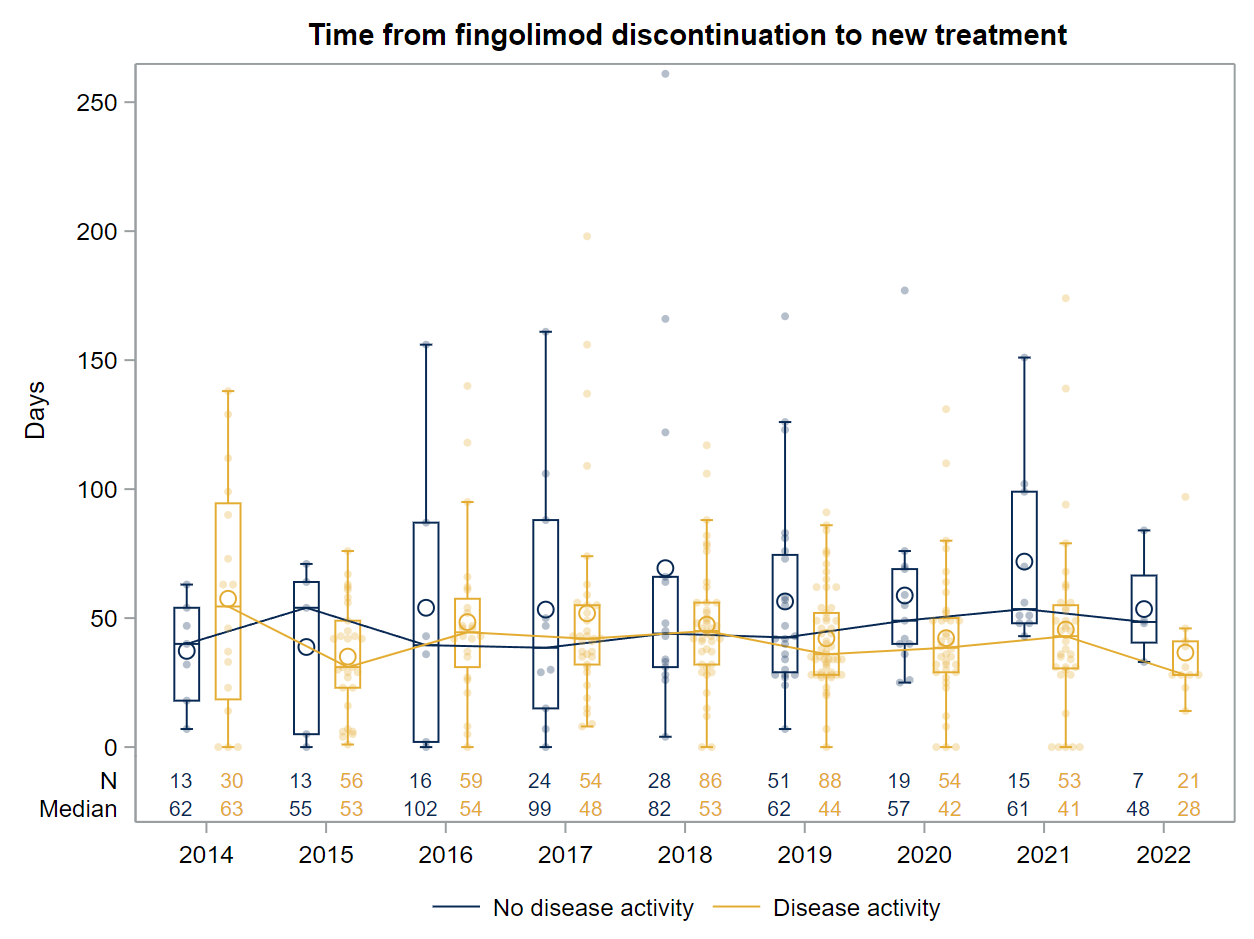


The experimental unit (N) is equal to the number of participants at each year. The median is the median number of days from fingolimod discontinuation to initiation of a new disease modifying therapy. The medians are connected with a line to illustrate trends. No statistical test was performed to produce the plot. Dots represent individuals. Patients were categorized as having disease activity if the reason for treatment discontinuation was reported as disease activity or if the patient had either a relapse or NT2L reported in the year leading up to the baseline date.

The graph is limited to individuals starting a new disease-modifying therapy within one year from fingolimod initiation.

**Supplementary Figure 2.** Days from fingolimod discontinuation to performance of the post-MRI.

**
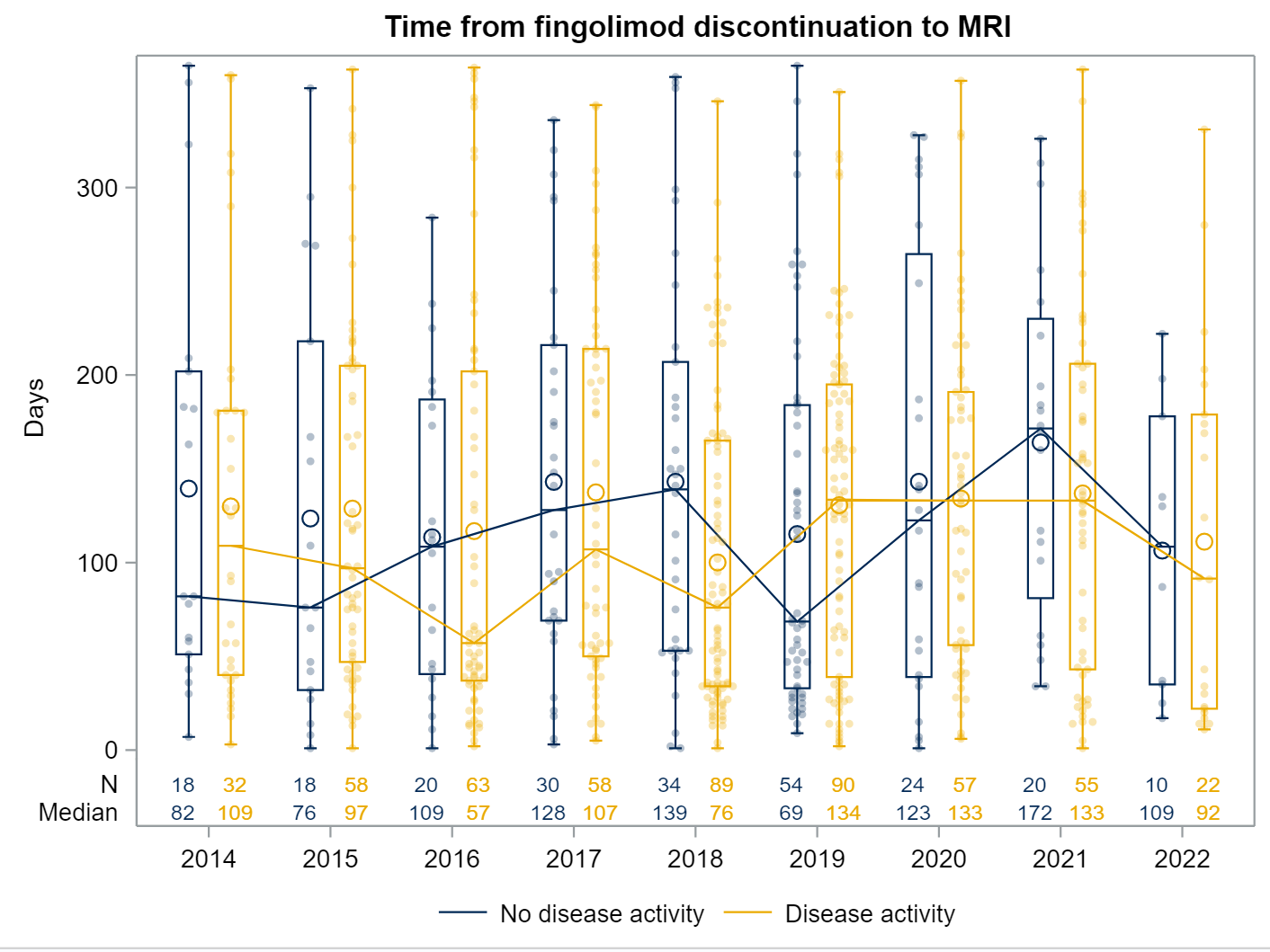
**

The experimental unit (N) is equal to the number of participants at each year. The median is the median number of days from fingolimod discontinuation to performance of the post-MRI. The medians are connected with a line to illustrate trends. No statistical test was performed to produce the plot. Dots represent individuals. Patients were categorized as having disease activity if the reason for treatment discontinuation was reported as disease activity or if the patient had either a relapse or NT2L reported in the year leading up to the baseline date.

**Supplementary Figure 3.** Flow diagram – dimethyl fumarate.


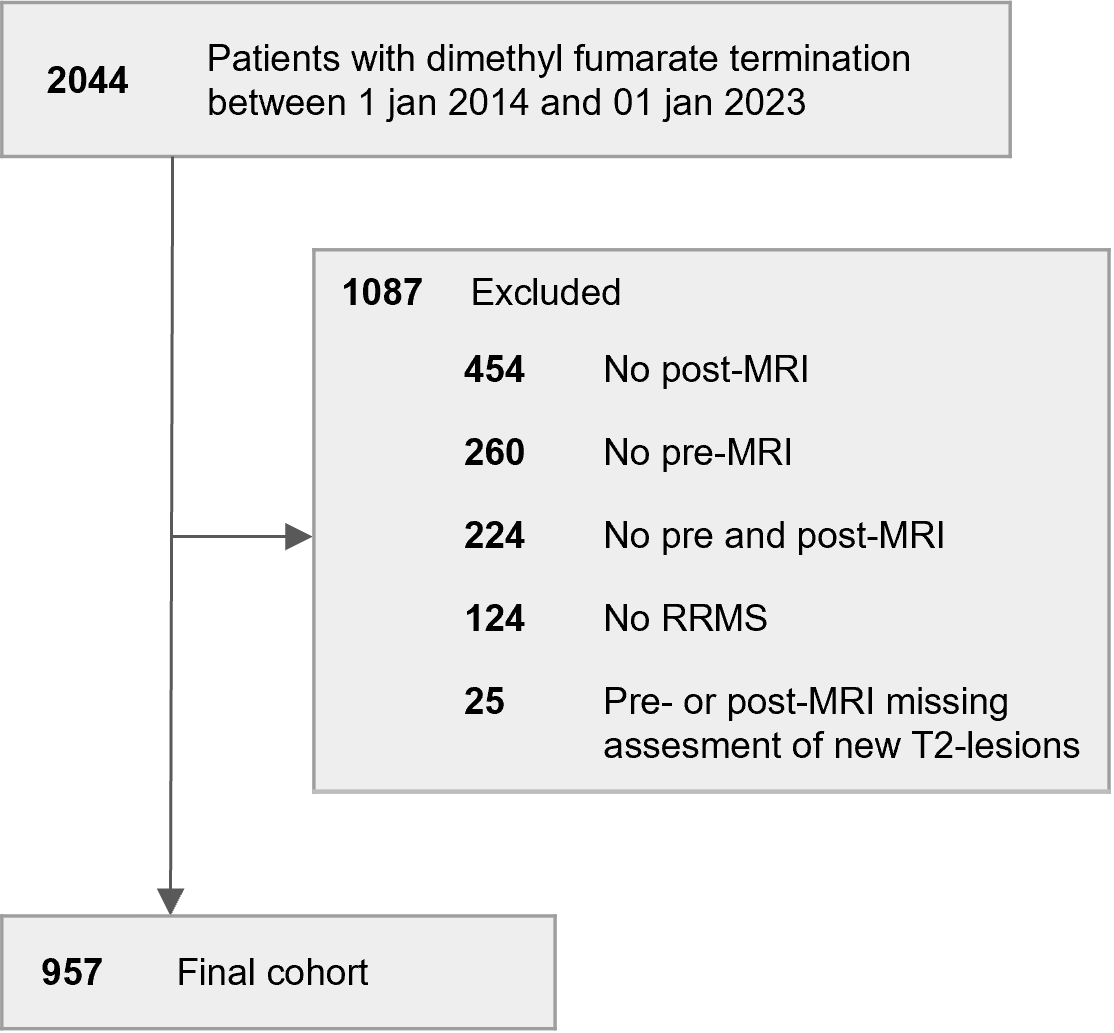


Definition of ‘pre-MRI’ and ‘post-MRI’: The patients had to have an MRI performed in the year leading up to the baseline date (the date of dimethyl fumarate termination), termed ‘pre-MRI’, and an MRI completed within one year after the baseline date, termed ‘post-MRI’.

RRMS: Relapsing-Remitting Multiple Sclerosis.

**Supplementary Table 2.** Baseline characteristics, unmatched, propensity score-matched and sensitivity analyses, fingolimod vs. dimethyl fumarate.

|  | | **Unmatched** | | **Propensity score-matched** | | **Propensity score-matched, sensitivity: lesion count^b^** | | **Propensity score-matched, sensitivity: new treatment efficacy and time to new treatment^c^** | |
| --- | --- | --- | --- | --- | --- | --- | --- | --- | --- |
|  | | **Fingolimod** | **Dimethyl fumarate** | **Fingolimod** | **Dimethyl fumarate** | **Fingolimod** | **Dimethyl fumarate** | **Fingolimod** | **Dimethyl fumarate** |
| **Clinical characteristics** | |  |  |  |  |  |  |  |  |
| Patients, No. | | 752 | 957 | 527 | 527 | 470 | 470 | 424 | 424 |
| Age at baseline, years, mean (SD) | | 41.4 (10.5) | 40.9 (11.0) | 40.8 (10.5) | 40.7 (11.0) | 40.9 (10.5) | 40.7 (11.0) | 41.1 (10.5) | 41.2 (11.1) |
| Sex, No. (%) | |  |  |  |  |  |  |  |  |
|  | Female | 552 (73.4) | 720 (75.2) | 389 (73.8) | 377 (71.5) | 348 (74.0) | 350 (74.5) | 303 (71.5) | 321 (75.7) |
|  | Male | 200 (26.6) | 237 (24.8) | 138 (26.2) | 150 (28.5) | 122 (26.0) | 120 (25.5) | 121 (28.5) | 103 (24.3) |
| Disease duration, years, mean (SD) | | 11.9 (7.6) | 8.4 (7.7) | 10.3 (7.1) | 10.5 (8.5) | 10.5 (7.2) | 10.3 (8.4) | 10.1 (7.0) | 10.4 (8.6) |
| EDSS, median (Q1-Q3) | | 2.5 (2.0-3.5) | 2.0 (1.0-3.0) | 2.5 (1.5-3.5) | 2.5 (2.0-3.5) | 2.5 (2.0-3.5) | 2.5 (1.5-3.5) | 2.5 (1.5-3.5) | 2.5 (1.5-3.5) |
| Number of relapses the past year, mean (SD) | | 0.7 (0.8) | 0.7 (0.7) | 0.6 (0.8) | 0.7 (0.7) | 0.7 (0.8) | 0.7 (0.7) | 0.6 (0.7) | 0.6 (0.7) |
| Treatment duration, years, median (Q1-Q3) | | 2.3 (0.9-4.5) | 1.0 (0.4-2.0) | 1.6 (0.6-3.2) | 1.2 (0.6-3.0) | 1.4 (0.6-2.9) | 1.2 (0.5-2.7) | 1.4 (0.5-2.8) | 1.1 (0.4-2.8) |
| Cause for treatment discontinuation, No. (%) | |  |  |  |  |  |  |  |  |
|  | Disease activity | 374 (49.7) | 385 (40.2) | 228 (43.3) | 256 (48.6) | 214 (45.5) | 203 (43.2) | 165 (38.9) | 162 (38.2) |
|  | Adverse events | 264 (35.1) | 410 (42.8) | 214 (40.6) | 204 (38.7) | 186 (39.6) | 193 (41.1) | 188 (44.3) | 184 (43.4) |
|  | Other^a^ | 70 (9.3) | 102 (10.7) | 51 (9.7) | 42 (8.0) | 42 (8.9) | 42 (8.9) | 43 (10.1) | 47 (11.1) |
|  | Pregnancy | 44 (5.9) | 60 (6.3) | 34 (6.5) | 25 (4.7) | 28 (6.0) | 32 (6.8) | 28 (6.6) | 31 (7.3) |
| **MRI characteristics** | |  |  |  |  |  |  |  |  |
| New T2-lesions on pre-MRI, No. (%) | |  |  |  |  |  |  |  |  |
|  | 0 | 472 (62.8) | 631 (65.9) | 333 (63.2) | 322 (61.1) | 301 (64.0) | 298 (63.4) | 283 (66.7) | 278 (65.6) |
|  | 1 | 112 (14.9) | 128 (13.4) | 79 (15.0) | 75 (14.2) | 74 (15.7) | 65 (13.8) | 54 (12.7) | 56 (13.2) |
|  | ≥ 2 | 168 (22.3) | 198 (20.7) | 115 (21.8) | 130 (24.7) | 95 (20.2) | 107 (22.8) | 87 (20.5) | 90 (21.2) |
| T2-lesion count on pre-MRI, No. (%) | |  |  |  |  |  |  |  |  |
|  | Missing | 72 (9.6) | 160 (16.7) | 41 (7.8) | 92 (17.5) | - | - | 24 (5.7) | 65 (15.3) |
|  | 0 | 336 (44.7) | 356 (37.2) | 246 (46.7) | 196 (37.2) | 243 (51.7) | 236 (50.2) | 209 (49.3) | 175 (41.3) |
|  | 1-19 | 118 (15.7) | 261 (27.3) | 93 (17.7) | 130 (24.7) | 98 (20.9) | 96 (20.4) | 71 (16.7) | 105 (24.8) |
|  | ≥ 20 | 226 (30.1) | 180 (18.8) | 147 (27.9) | 109 (20.7) | 129 (27.5) | 138 (29.4) | 120 (28.3) | 79 (18.6) |
| Days since pre-MRI, days, median (Q1-Q3) | | 98 (45-193) | 98 (45-191) | 99 (46-196) | 93 (45-191) | 102 (47-193) | 99 (48-201) | 102 (48-198) | 115 (49-205) |
| Days between pre-MRI and previous comparison MRI, median (Q1-Q3) | | 344 (194-409) | 300 (171-447) | 324 (183-394) | 319 (175-466) | 298 (177-386) | 330 (180-477) | 298 (183-391) | 338 (186-497) |
| **New DMT characteristics** | |  |  |  |  |  |  |  |  |
| DMT initiated at post-MRI, No. (%) | |  |  |  |  |  |  |  |  |
|  | None | 373 (49.6%) | 311 (32.5%) | 273 (51.8%) | 167 (31.7%) | 242 (51.5%) | 167 (35.5%) | 216 (50.9%) | 202 (47.6%) |
|  | Moderate efficacy | 64 (8.5%) | 237 (24.8%) | 52 (9.9%) | 101 (19.2%) | 47 (10.0%) | 96 (20.4%) | 53 (12.5%) | 58 (13.7%) |
|  | High efficacy | 315 (41.9%) | 409 (42.7%) | 202 (38.3%) | 259 (49.1%) | 181 (38.5%) | 207 (44.0%) | 155 (36.6%) | 164 (38.7%) |
| Time to treatment (if new DMT initiated), No. (%) | |  |  |  |  |  |  |  |  |
|  | None | 373 (49.6%) | 311 (32.5%) | 273 (51.8%) | 167 (31.7%) | 252 (51.5%) | 167 (35.5%) | 216 (50.9%) | 202 (47.6%) |
|  | ≤ 30 days | 104 (13.8%) | 449 (46.9%) | 67 (12.7%) | 260 (49.3%) | 64 (13.6%) | 215 (45.7%) | 89 (21.0%) | 101 (23.8%) |
|  | > 30 days | 275 (36.6%) | 197 (20.6%) | 187 (35.5%) | 100 (19.0%) | 164 (34.9%) | 88 (18.7%) | 119 (28.1%) | 121 (28.5%) |

^a^ Listed as other: lack of patient compliance, practical issues, stable condition, contra-indication, or patient decision. ^b^ Additionally matched on T2-lesion load on pre-MRI (0, 1-19 or ≥ 20). ^c^ Additionally matched on treatment status at post-MRI according to treatment efficacy (none, moderately or highly effective disease modifying therapy) and new treatment initiation ≤ 30 days from fingolimod/dimethyl fumarate termination or > 30 days. EDSS: Expanded Disability Status Scale, DMT: Disease-modifying therapy.

New disease-modifying therapies initiated after fingolimod/dimethyl fumarate termination and before the post-MRI were categorized into moderately and highly effective for the sensitivity analysis according to the following table:

| Moderate efficacy | Teriflunomide, interferon beta-1a, interferon beta-1b, peginterferon beta-1a, glatiramer acetate, azathioprine, dimethyl fumarate, diroximel fumarate |
| --- | --- |
| High efficacy | Ofatumumab, hematopoietic stem cell transplantation, alemtuzumab, rituximab, cladribine, methotrexate, mitoxantrone, ocrelizumab, fingolimod, natalizumab, daclizumab, Siponimod, ozanimod |

**Supplementary Table 3.** Baseline characteristics of patients excluded due to missing pre-MRI, post-MRI or both pre- and post-MRI.

|  | **Fingolimod** | | **Dimethyl fumarate** | |
| --- | --- | --- | --- | --- |
|  | **Included** | **Excluded** | **Included** | **Excluded** |
| Patients, No. | 752 | 419 | 957 | 963 |
| **Clinical characteristics** |  |  |  |  |
| Age at baseline, years, mean (SD) | 41.4 (10.5) | 41.7 (10.9) | 40.9 (11.0) | 42.5 (11.5) |
| Sex, No. (%) |  |  |  |  |
| Female | 552 (73.4) | 285 (68.0) | 720 (75.2) | 775 (80.5) |
| Male | 200 (26.6) | 134 (32.0) | 237 (24.8) | 188 (19.5) |
| Disease duration, years, mean (SD) | 11.9 (7.6) | 12.3 (7.5) | 8.4 (7.7) | 10.6 (8.0) |
| EDSS, median (Q1-Q3) | 2.5 (2.0-3.5), *n_miss_*=38 | 2.5 (1.5-4.0), *n_miss_*=66 | 2.0 (1.0-3.0), *n_miss_*=86 | 2.0 (1.5-3.0), *n_miss_*=129 |
| Number of relapses in the past year, mean (SD) | 0.7 (0.8) | 0.4 (0.7) | 0.7 (0.7) | 0.3 (0.6) |
| Treatment duration, years, median (Q1-Q3) | 2.3 (0.9-4.5) | 2.4 (1.1-4.7) | 1.0 (0.4-2.0) | 1.0 (0.4-2.1) |
| Recorded cause for treatment discontinuation, No. (%) |  |  |  |  |
| Disease activity | 374 (49.7) | 116 (27.7) | 385 (40.2) | 162 (16.8) |
| Adverse events | 264 (35.1) | 145 (34.6) | 410 (42.8) | 494 (51.3) |
| Other^a^ | 70 (9.3) | 102 (24.3) | 102 (10.7) | 155 (16.1) |
| Pregnancy wish | 44 (5.9) | 56 (13.4) | 60 (6.3) | 152 (16.8) |
| Disease activity^b^, No. (%) | 524 (69.7) | 180 (43.0) | 666 (69.6) | 374 (38.8) |
| **MRI characteristics** |  |  |  |  |
| Pre-MRI, No. (%) | - | 213 (50.8) | - | 456 (47.4) |
| New T2-lesions on pre-MRI, grouped, No. (%) |  |  |  |  |
| 0 | 472 (62.8) | 160 (75.1) | 631 (66.0) | 324 (71.1) |
| 1-2 | 171 (22.7) | 35 (16.4) | 212 (22.2) | 95 (20.8) |
| ≥ 3 | 109 (14.5) | 18 (8.5) | 114 (11.9) | 37 (8.1) |
| Time from pre-MRI to baseline, days, median (Q1-Q3) | 98 (45-193) | 106 (50-193) | 98 (45-191) | 110 (46-216) |
| Post-MRI, No. (%) | - | 117 (27.9) | - | 266 (27.6) |
| New T2-lesions on post-MRI, grouped, No. (%) |  |  |  |  |
| 0 | 501 (66.6) | 83 (70.9) | 798 (83.4) | 210 (79.0) |
| 1-2 | 127 (16.9) | 25 (21.4) | 114 (11.9) | 38 (14.3) |
| ≥ 3 | 124 (16.5) | 9 (7.7) | 45 (4.7) | 18 (6.8) |
| Time from baseline to post-MRI, days, median (Q1-Q3) | 111 (41-195) | 91 (36-180) | 151 (81-233) | 109 (41-217) |

^a^ Listed as other: lack of patient compliance, practical issues, stable condition, contra-indication, or patient decision.

^b^ Status was defined as “disease activity” if the patient met one of the following criteria: fingolimod discontinuation due to disease activity, one or more relapses in the last year before fingolimod discontinuation, or the presence of new T2-lesion(s) on the last MRI in the last year before fingolimod discontinuation. Note: Some of the excluded patients will not have had an MRI performed in the last year before fingolimod discontinuation.

EDSS: Expanded Disability Status Scale.

**Supplementary Table 4.** Frequency of new T2-lesions on post-MRI, sensitivity analysis.

|  | | **Fingolimod** | **Probability (95% CI)^a^** | **Dimethyl fumarate** | **Probability (95% CI)^a^** | **Odds ratio^b^** |
| --- | --- | --- | --- | --- | --- | --- |
| **UNMATCHED** | |  |  |  |  |  |
| Patients, No. | | 752 |  | 957 |  |  |
| New T2-lesions, No. | | |  |  |  |  |
|  | 0 | 501 |  | 798 |  |  |
|  | 1-2 | 127 | 16.9% (14.2-19.6) | 114 | 11.9% (9.9-14.0) | 1.8 (1.3-2.3)^c^ |
|  | ≥ 3 | 124 | 16.5% (13.8-19.1) | 45 | 4.7% (3.4-6.0) | 4.4 (3.1-6.3)^c^ |
| **MATCHED^d^** | | | |  |  |  |
| Patients, No. | | 527 |  | 527 |  |  |
| New T2-lesions, No. | | | |  |  |  |
|  | 0 | 343 |  | 444 |  |  |
|  | 1-2 | 96 | 18.2% (14.9-21.5) | 60 | 11.4% (8.7-14.1) | 2.1 (1.5-2.9)^c^ |
|  | ≥ 3 | 88 | 16.7% (13.5-19.9) | 23 | 4.4% (2.6-6.1) | 5.0 (3.1-8.0)^c^ |
| **SENSITIVITY: LESION COUNT** | | | |  |  |  |
| Patients, No. | | 470 |  | 470 |  |  |
| New T2-lesions, No. | |  |  |  |  |  |
|  | 0 | 308 |  | 388 |  |  |
|  | 1-2 | 83 | 17.7% (14.2-21.1) | 57 | 12.1% (9.2-15.1) | 1.8 (1.3-2.7)^c^ |
|  | ≥ 3 | 79 | 16.8% (13.4-20.2) | 25 | 5.3% (3.3-7.3) | 4.0 (2.5-6.4)^c^ |
| **SENSITIVITY: NEW TREATMENT EFFICACY AND TIME TO NEW TREATMENT** | | | |  |  |  |
| Patients, No. | | 424 |  | 424 |  |  |
| New T2-lesions, No. | |  |  |  |  |  |
|  | 0 | 288 |  | 355 |  |  |
|  | 1-2 | 72 | 17.0% (13.4-20.6) | 50 | 11.8% (8.7-14.9) | 1.8 (1.2-2.6), *p* = 0.004 |
|  | ≥ 3 | 64 | 15.1% (11.7-18.5) | 19 | 4.5% (2.5-6.5) | 4.2 (2.4-7.1)^c^ |

^a^ Predictions from the multinominal logistic regression, see method section.

^b^ Fingolimod vs dimethyl fumarate.

^c^ *P* <.001

^d^ Propensity score matched based on age, sex, Expanded Disability Status Scale, number of new T2-lesions on pre-MRI, cause for termination of treatment, treatment duration, number of relapses the past year and an interaction between age and sex.
